# Mammary analogue secretory carcinoma of salivary glands: a new entity associated with *ETV6* gene rearrangement

**DOI:** 10.1007/s00428-014-1701-8

**Published:** 2014-12-12

**Authors:** Hanna Majewska, Alena Skálová, Dominik Stodulski, Adéla Klimková, Petr Steiner, Czesław Stankiewicz, Wojciech Biernat

**Affiliations:** 1 Department of Pathomorphology, Medical University of Gdańsk, Gdańsk, Poland; 2 Department of Pathology, Faculty of Medicine in Plzen, Charles University in Prague, Prague, Czech Republic; 3 Department of Otolaryngology, Medical University of Gdańsk, Gdańsk, Poland; 4 Bioptic Laboratory, Ltd., Molecular Pathology Laboratory, Plzen, Czech Republic; 5 Department of Pathology, Medical University of Gdansk, ul.Smoluchowskiego, 80-211 Gdańsk, Poland

**Keywords:** Mammary analogue secretory carcinoma, MASC, Salivary gland, *ETV6*-*NTRK3* fusion, Translocation t(12;15)

## Abstract

Mammary analogue secretory carcinoma (MASC) is a recently described salivary gland tumour that harbours the recurrent *ETV6*-*NTRK3* translocation. This is the first series of MASC cases identified in the historic cohort of carcinomas of salivary glands with clinical/pathological correlation and follow-up data. We reviewed 183 primary carcinomas of major and minor salivary glands resected at the Medical University of Gdańsk, Poland, between 1992 and 2012. Based on morphology and immunohistochemistry, cases suspicious for MASC were selected, and the diagnosis was confirmed by fluorescence in situ hybridization (FISH) for *ETV6* rearrangement and by RT-PCR for the *ETV6*-*NTRK3* fusion transcript. Seven carcinomas met the criteria of MASC, as they exhibited a typical appearance with solid/microcystic and papillary architecture and intraluminal secretions, and cells completely devoid of basophilic cytoplasmic zymogen granules indicative of true acinar differentiation. The only paediatric case was an unencapsulated tumour composed of macrocystic structures covered by a mostly single but, focally, double layer of cells with apocrine morphology. In all cases, the neoplastic cells revealed immunoreactivity for S100, mammaglobin, cytokeratin CK7, CK8, STAT5a and vimentin. FISH for *ETV6* gene rearrangement was positive in six out of seven cases, and RT-PCR was positive in three cases. MASC is a new entity of malignant epithelial salivary gland tumours not included in the 2005 WHO Classification of Head and Neck Tumours. There is a growing body of evidence that it is not as rare as was assumed, as is also indicated by our series (3.8 %). In most cases, MASC shares some microscopic features with AciCC, adenocarcinoma/cystadenocarcinoma NOS and low-grade MEC. In rare cases, MASC with high-grade transformation may mimic the morphological appearances of high-grade salivary gland malignancies, such as salivary duct carcinoma.

## Introduction

Mammary analogue secretory carcinoma (MASC) is a new tumour entity described by Skálová et al. in 2010 [26] that harbours the recurrent translocation t(12;15)(p13;q25) resulting in the *ETV6*-*NTRK3* gene fusion, the same cytogenetic abnormality is described earlier in secretory breast carcinoma [26, 27]. The fusion gene *ETV6*-*NTRK3* encodes a chimeric tyrosine kinase, which has potential transforming activity and plays a major role in oncogenesis [8]. Conceivably, a small molecular tyrosine kinase inhibitor might be a potential treatment for patients of whom the tumour carries this fusion gene [8]. The resultant fusion protein *ETV6*-*NTRK3* has transforming activity, not only in epithelial but also in mesenchymal and blood cell lineages. Earlier, the *ETV6*-*NTRK3* translocation has been described in infantile fibrosarcoma [16], congenital mesoblastic nephroma and acute myelogenous leukaemia [8, 16]. *ETV6* is genetically unstable and fuses not only with *NTRK3* but also with other genes such as *ABL1*, *EGFR3*, *PAX5*, *SYK* and *JAK2* in leukaemia, myelodysplastic syndromes and sarcomas [8, 27].

Since the seminal paper of Skálová et al. [26], a number of retrospective studies and case reports have been published. They further characterized the tumour in terms of histopathology and immunohistochemistries [3, 9, 13, 15, 18, 22] as well as cytology [6, 14, 19]. However, the number of large clinico-pathological studies with long follow-up data describing the full spectrum of salivary gland tumours that may mimic MASC is very limited. Single studies have re-evaluated historical files of acinic cell carcinomas (AciCC) [13, 18] or other classical mimickers in the light of this newly emerging entity [10]. Only a single study of a historical retrospective cohort of the whole spectrum of salivary gland tumours has been published so far [15].

Histomorphologically, MASC is a distinctive entity [26], and histology in conjunction with an appropriate immunohistochemical profile is sufficient for a diagnosis in most cases. However, several histomorphological features of MASC overlap with those of other salivary gland tumours [24, 26, 29]. AciCC and adenocarcinomas/cystadenocarcinomas NOS are the most frequent MASC mimics, followed by low-grade mucoepidermoid carcinoma [3, 24]. The aim of our study is to describe the morphological and clinical features of MASC in seven patients identified retrospectively from a variety of low- and high-grade malignant epithelial salivary gland tumours.

## Materials and methods

We reviewed all the primary carcinomas of major and minor salivary glands (183) resected at the Medical University of Gdańsk (Departments of Otolaryngology and Maxillofacial Surgery) between 1992 and 2012 and reclassified them according to the criteria published by WHO 2005 (HM and AS) [2] based on histomorphology and immunohistochemistry. In cases suspicious for MASC, fluorescence in situ hybridization (FISH) for detection of *ETV6* rearrangement was performed.

The salivary gland tumour material included adenoid cystic carcinoma, (AdCC, *n* = 61), mucoepidermoid carcinoma (MEC, *n* = 25), carcinoma ex pleomorphic adenoma (CXPA, *n* = 24), acinic cell carcinoma (AciCC, *n* = 17), adenocarcinoma (*n* = 14), salivary duct carcinoma (SDC, *n* = 11), polymorphous low-grade adenocarcinoma (PLGA, *n* = 7), epithelial-myoepithelial carcinoma (*n* = 6), basal cell carcinoma (*n* = 4), undifferentiated carcinoma (*n* = 3), squamous cell carcinoma (*n* = 3), myoepithelial carcinoma (*n* = 2), neuroendocrine carcinoma (*n* = 2), papillary cystadenocarcinoma (*n* = 2), lymphoepithelial carcinoma (*n* = 1) and one case of newly recognized entity of cribriform adenocarcinoma of the tongue and other minor salivary glands (CATS). Based on histomorphology and expression of immunohistochemical markers, seven cases of mammary analogue secretory carcinoma (MASC) were retrieved. The original diagnoses in these cases include AciCC (two cases), adenocarcinoma (two cases), cystadenocarcinoma, MEC and SDC (one case each).

Paraffin blocks and recuts were available for histological and immunohistochemical analysis for all the studied cases. Clinical data and follow-up were obtained from the patients or their physicians (DS, CS).

## Immunohistochemical study

For conventional microscopy, resected tissues were cut and stained with haematoxylin and eosin. For immunohistochemistry, 4-μm-thick sections were cut from paraffin blocks, mounted on silanized slides, deparaffinized in xylene and rehydrated in descending grades (100–70 %) of ethanol. Sections were then subjected to heat-induced epitope retrieval by immersion in a 0.01 citrate buffer at pH 6 at 95 °C in a microwave oven (Micromed TTmega) for 20 min. Endogenous peroxidase was blocked by a 5-min treatment with 3 % hydrogen peroxide in absolute methanol. The slides were then stained by immunostainer BenchMark ULTRA (Roche). The primary antibodies employed in the study are listed in Table 1. The bound antibodies were visualized using the Histofine Simple Stain MAX PO (Multi) Universal Immuno-peroxidase Polymer, Anti-Mouse and Rabbit (Nichirei Biosciences inc., Tokyo, Japan), and 3-3′-diaminobenzidine (Sigma) as chromogen. The slides were counterstained with Mayer’s haematoxylin. Appropriate positive and negative controls were employed.

**Table 1:** Antibodies used and sources

Antibody	Clone	Dilution	Source
CK7	OV-TL 12/30	1:200	Dako
CK8	35 βH11	RTU	Dako
S-100 protein	Polyclonal	1:2000	Dako
Mammaglobin	304-1A5	RTU	Dako
STAT5	Polyclonal	1:400	Assay designs
P63	4A4	RTU	Ventana
P40	N/A	RTU	Roche
Vimentin	V9	RTU	Dako
DOG1	Polyclonal	RTU	Roche

VENTANA at pH 8 and at 95 °C

*RTU* ready to use

## Molecular genetic study

### Detection of the ETV6-NTRK3 fusion transcript by RT-PCR

RNA from all cases of MASCs was extracted using the RecoverAll Total Nucleic Acid Isolation Kit (Ambion, Austin, TX, USA). Synthesis of complementary DNA (cDNA) was performed using the Transcriptor First Strand cDNA Synthesis Kit (RNA input 1 μg) (Roche Diagnostics, Mannheim, Germany). All procedures were performed according to the manufacturer’s protocols. Amplification of the 105 and 133 bp product of the two-microglobulin gene and the 247-bp product of the *PGK* gene was used to test the quality of the extracted RNA, as previously described [1, 11, 28].

Detection of 110 bp fragments of *ETV6*-*NTRK3* fusion transcripts was performed by RT-PCR, as follows [7]. Two microliters of cDNA was added to a reaction mixture containing 12.5 μl of Hot Star Taq PCR Master Mix (QIAgen, Hilden, Germany), 10 pmol of each primer TRKC1059 complementary to *NTRK3* with sequence (5′-CAGTTCTCGCTTCAGCACGATG-3′) and TEL971 complementary to *ETV6* with sequence (5′-ACCACATCATGGTCTCTGTCTCCC-3′) and distilled water up to 25 μl. The amplification program comprised of denaturation at 95 °C for 14 min and 45 cycles of denaturation at 95 °C for 1 min, annealing at 65 °C for 1 min and extension at 72 °C for 1 min. The program was finished by incubation at 72 °C for 7 min.

Successfully amplified PCR products were purified with magnetic beads Agencourt® AMPure® (Agencourt Bioscience Corporation, A Beckman Coulter Company, Beverly, MA, USA). The products were then bi-directionally sequenced using the Big Dye Terminator Sequencing kit (PE/Applied Biosystems, Foster City, CA, USA) purified with magnetic particles Agencourt® CleanSEQ® (Agencourt Bioscience Corporation), all according to the manufacturer’s protocol and run on an automated sequencer ABI Prism 3130xl (Applied Biosystems, Foster City, CA, USA) at a constant voltage of 13.2 kV for 11 min.

## Detection of ETV6-NTRK3 gene break by FISH

### FISH method

For the FISH study, the LSI ETV6 (TEL) Dual Color, Break Apart Rearrangement Probe (VYSIS/Abott, Abott Park, IL) was used. The specimen, a 4-μm-thick FFPE section, was placed onto a positively charged slide. Tissues were deparaffinized in xylene three times for 5 min and then washed twice in 100 % ethanol once in 95 % ethanol and once in deionized water for 5 min. The slides were then heated in the 1× Target Retrieval Solution (pH 6) (DAKO, Glostrup, Denmark) for 40 min at 95 °C and subsequently cooled for 20 min at room temperature in the same solution. The slides were washed in deionized water for 5 min and covered with the Proteinase K (20 mg/ml) (SERVA, Heidelberg, Germany) for 10 min at room temperature. The slides were then placed into deionized water for 5 min, dehydrated in a series of ethanol solution (70, 85 and 96 % for 2 min each) and air-dried. An appropriate amount of FISH probe was applied onto each specimen, which was then covered with a glass cover slip and sealed with rubber cement. The slides were incubated in the ThermoBrite^TM^ instrument (StatSpin/Iris Sample Processing, Westwood, MA, USA) with co-denaturation parameters at 85 °C for 8 min and hybridization parameters 37 °C for 16 h. The rubber cemented cover slips were then removed, and the slides were placed in a post-hybridization wash solution (2xSSC/0.3 % NP-40) at 72 °C for 2 min. The slides were air-dried in the dark, counterstained with DAPI II (VYSIS/Abbott), cover slipped and immediately examined.

### FISH interpretation

Hybridized slides were examined with an Olympus BX51 fluorescence microscope using a ×100 objective and as filter sets triple band pass (DAPI/Spectrum Green/Spectrum Orange), dual band pass (FITC/Texas Red) and single band pass (Spectrum Green or Spectrum Orange) filters. One hundred randomly selected non-overlapping tumour cell nuclei were examined for the presence of yellow (normal) or green and red (chromosomal breakpoint) fluorescent signals. The sample was considered positive if more than 10 % of nuclei showed a breakpoint signal. Molecular genetic analysis (RT-PCR and FISH) was performed in Biopticka Laboratory in Plzen, Czech Republic (AK, PS).

## Results

### Clinical and follow-up data

The seven patients with MASC concerned two females and five males ranging in age between 17 and 73 years (median 51.4 years). One tumour was located in the hard palate; the other six were in the parotid gland. The duration of symptoms was known for five of seven patients, on the average 16.2 months (range 3–38 months). For four patients, the clinical course was indolent with a non-tender slowly growing mass covered by intact skin (three tumours in the parotid gland) or mucous membrane (one tumour in the palate). The other three patients presented with symptoms suggesting malignancy, such as accelerated growth, pain, skin infiltration, neck lymphadenopathy or ulceration of the skin or a mucous membrane. Four patients presented in early clinical stage (I or II), and three patients were in stages III and IVa. The clinical features are summarized in Table 2.

**Table 2:** Clinico-pathological features of patients with mammary analogue of secretory carcinoma

	Sex/age	Localization	Tumour size (cm)	Clinical course	TNM/Stage (2002)	Treatment	Surgical margins/LN metastases	Status/months
1	F/42	Parotid	2.2 × 1.3	Mild (3 months), asymptomatic cystic mass	T2N0/II	PCP	Negative	NED/96
2	M/63	Parotid	2.3 × 2.0	Aggressive (12 months) mass with rapid growth, pain, skin infiltration, neck lymphadenopathy	T3N1/III	PCP, MRND RT	Close LN metastases (−)	NED/90
3	F/51	Parotid	2.5 × 1.2	Mild (24 months) asymptomatic cystic mass	T2N0/II	PCP	Close	NED/67
4	M/17	Parotid	4 × 3.5 × 2.5	Mild, multinodular tumour	T2N0/II		Negative	NED/120
5	M/73	Parotid	3 × 1.5 × 1.4	Aggressive, neck lymphadenopathy, skin infiltration, facial nerve paralysis, 6× reoperated due to lymph node meta or local recurrences	T2N2b/IVa	RT, ChT	Positive/multiple LN metastasis	DOD/79
6	M/60	Parotid	4 × 3.5 × 2.5	Aggressive (6 months) mass with rapid growth, skin infiltration, neck lymphadenopathy	T4aN2b/IVa	TSCP, SND RT	Positive LN metastases (+) 7/7 ECS	Local recurrence/4–excision distant metastases (lungs, bones)/16 DOD/20
7	M/54	Hard palate	2.0 × 1.0	Mild (36 months) > aggressive (2 months) mass smooth > ulcerated mucous membrane	T1N0/I	HP resection	Close	Loco-regional recurrence/48 lateral rhinotomy, SND, RT LN metastases (+) 1/11; Surgical margins (+) NED/31 (overall survival/79)

*FNAB* fine needle aspiration biopsy, *PCP* partial conservative parotidectomy, *TSCP* total semi-conservative parotidectomy, *SND* selective neck dissection, *MRND* modified radical neck dissection, *RT* radiation therapy, *HP* hard palate, *AciCC* acinic cell carcinoma, *MEC* mucoepidermoid carcinoma, *NED* no evidence of disease, *DOD* died on disease

Fine needle aspiration biopsy (FNAB) results are summarized in Table 3. In two cases, the tumours were preoperatively diagnosed as benign lesions (cyst and adenoma) and in three cases as malignancy. All patients underwent surgical treatment: partial conservative parotidectomy (PCP, with facial nerve preservation) was performed in five patients and semi-conservative (PSCP, with preservation of some facial nerve branches) in one patient. In one case, partial resection of the hard palate was performed. In two cases, the neck lymph nodes were dissected. In three of seven cases, patients received supplementary radiotherapy due to metastases to the regional lymph nodes and/or positive or uncertain surgical resection margins. One patient was treated by chemotherapy (no. 5).

**Table 3:** Results of fine needle aspiration biopsy, original histological diagnosis of patients, RT-PCR and FISH results

	Sex/age	Original cytopathologic diagnosis	Original histologic diagnosis	RT-PCR	FISH	Final diagnosis
1	F/42	Cyst	Low-grade cystadenocarcinoma NOS	Negative	Not diagnostic	MASC
2	M/62	Adenocarcinoma	Adenocarcinoma NOS, grade 2	Negative	Positive [38/100]	MASC
3	F/51	Adenoma	AciCC /papillary cystic variant	Positive	Positive [81/100]	MASC
4	M/17	–	AciCC	Negative	Positive [39/100]	MASC
5	M/73	–	SDC grade 2	Positive	Positive [80/100]	MASC with high-grade transformation
6	M/60	Adenocarcinoma	MEC grade 2	Negative	Positive [89/100]	MASC with high-grade transformation
7	M/54	LG carcinoma of salivary gland	Adenocarcinoma NOS, grade 2	Positive	Positive [74/100]	MASC

Four of seven patients (cases 1–4) remained without evidence of disease during 67–120 months follow-up (median 93 months). In one case (no. 7), loco-regional recurrence occurred 48 months after excision of the hard palate tumour. This patient remained disease-free after combined treatment (lateral rhinotomy with neck dissection and radiation therapy) for 31 months. Two patients (cases five and six), who were also described elsewhere [25], died of disease progression 20 and 79 months after diagnosis.

### Histopathological and immunohistochemical findings

**Fig. 1:**
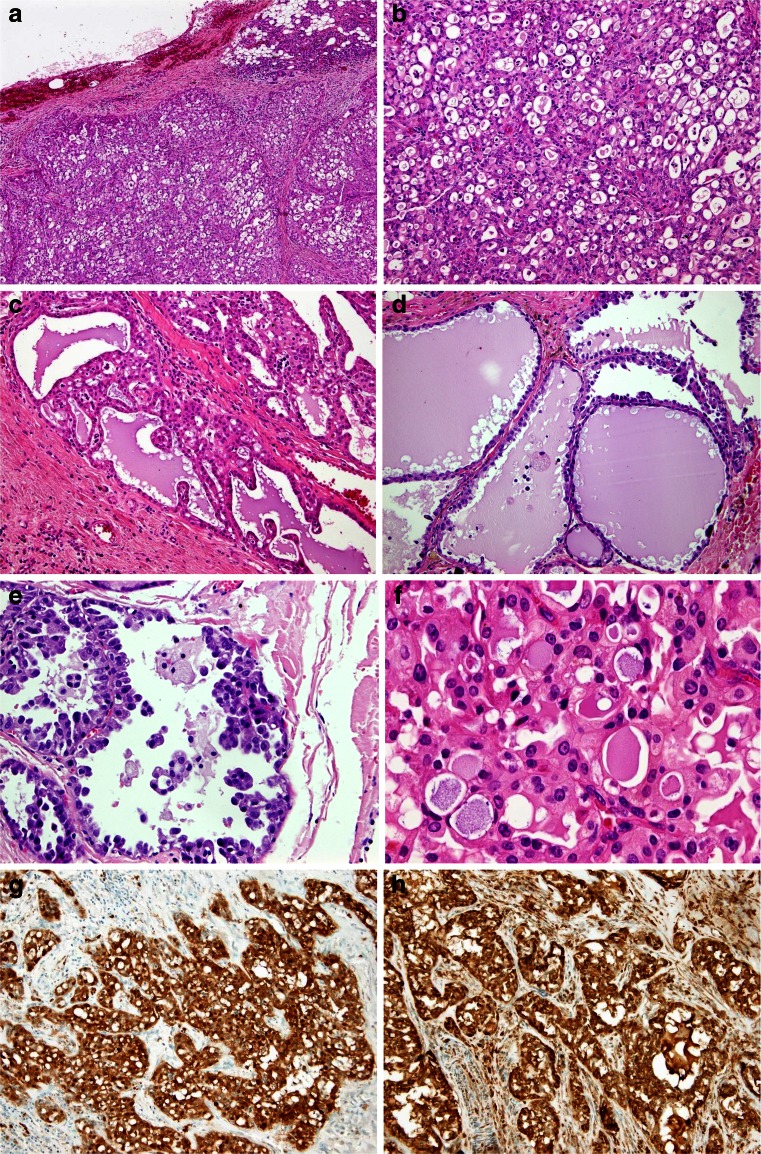
Histopathological features of MASC: **a** the tumour is well circumscribed and surrounded by a thick, not interrupted fibrous capsule (H&E; ×40); **b** microcystic and slightly dilated glandular spaces filled with an eosinophilic homogenous secretory material (H&E; ×100); **c** minor component is represented by papillary structures (H&E; ×100); **d** a macrocystic growth pattern (H&E; ×100); **e** cystic structures lined mostly by a single and, focally, a double layer of cells with focal apocrine differentiation (H&E; ×200); **f** cells with abundant pale pink vacuolated and foamy cytoplasm and vesicular, bland looking nuclei with prominent nucleoli (H&E; ×200); **g** a diffuse and strong staining for S100 and **h** mammaglobin (×100)

On low power magnification, MASC displayed three major growth patterns. Firstly, some tumours were well circumscribed and surrounded by a thick, uninterrupted fibrous capsule (cases 1 and 3) with predominating papillary and microcystic structures (Fig. 1a). In the second pattern, the tumour revealed a solid and lobular growth pattern characterized by a multilobular structure divided by hyalinized or fibrous septa with local infiltrative borders, unencapsulated or only partially encapsulated (cases 2 and 5–7). These cases were predominantly composed of microcystic and slightly dilated glandular spaces filled with an eosinophilic homogenous secretory material (Fig. 1b). A minor component was represented by some papillary structures (Fig. 1c). The third pattern in the only paediatric case (no. 4) was macrocystic (Fig. 1d). The tumour appeared unencapsulated and was composed of cystic structures lined mostly by a single and focally a double layer of cells with focal apocrine differentiation (Fig. 1e). The cysts contained abundant protein-like eosinophilic material. The tumour cells revealed abundant pale pink vacuolated and foamy cytoplasm with vesicular, bland-looking nuclei and prominent nucleoli (Fig. 1 f).

**Fig. 2:**
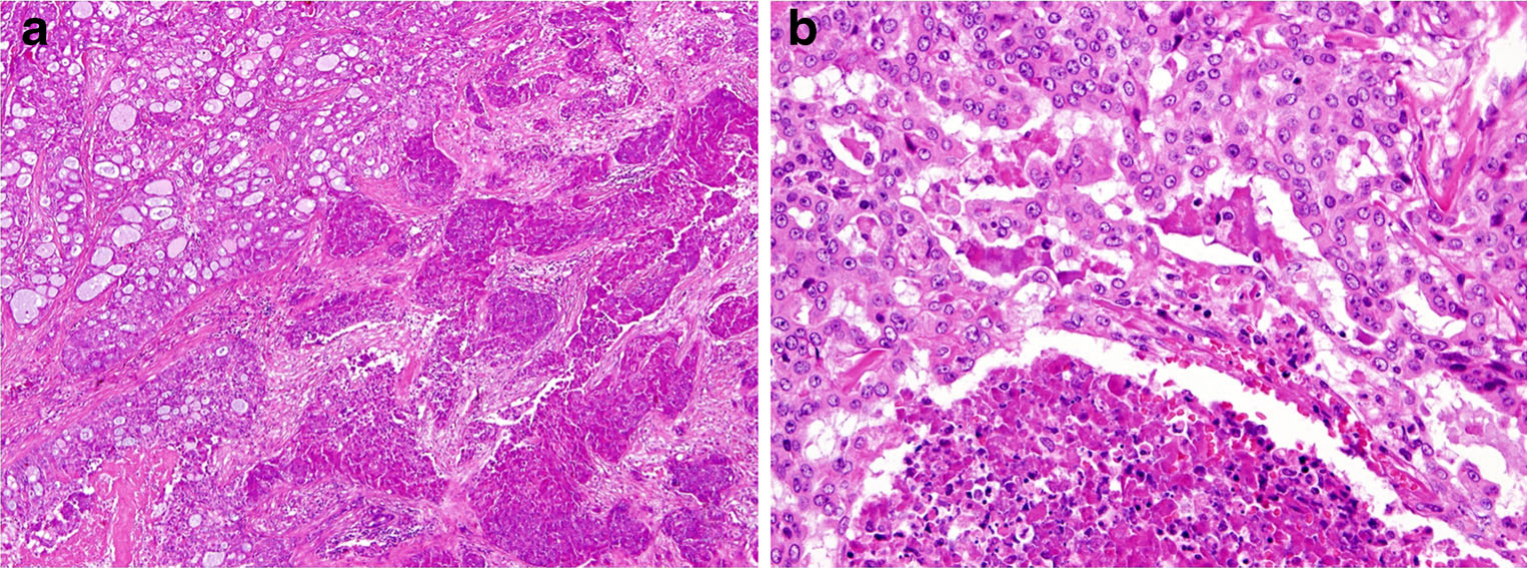
MASC with high-grade (HG) transformation: **a** The tumour contains two distinct carcinomatous components. One represents conventional MASC composed of uniform neoplastic cells arranged in solid, tubular and microcystic growth structures divided by fibrous septa that were partly hyalinized. The tumour cells show typical low-grade morphology: vesicular round to oval nuclei with finely granular chromatin and distinct centrally located nucleoli (*left*). The HG component is composed of anaplastic cells with abundant cytoplasm and large pleomorphic nuclei (*right*) (H&E; ×40); **b** solid tumour islands of MASC high-grade component with areas of large geographical comedo-like necrosis (H&E; ×200)

In two cases (cases 5 and 6), the tumours were composed of two distinct carcinomatous components (Fig. 2). One component was a conventional MASC composed of uniform neoplastic cells arranged in solid, tubular and microcystic structures divided by fibrous septa that were partly hyalinized. The tumour cells had typical low-grade morphology with vesicular round to oval nuclei with finely granular chromatin and distinct centrally located nucleoli (Fig. 2a). The other component, sharply delineated from the conventional MASC, was of high grade (HG) and composed of anaplastic cells with abundant cytoplasm and large pleomorphic nuclei. Solid tumour islands revealed areas of large geographical comedo-like necrosis (Fig. 2b). Desmoplastic stroma was indicative of invasion. Tumour cells of the HG component had high mitotic activity and nuclear polymorphism and failed to produce secretory material in contrast to the low-grade component of MASC. Perineural invasion was observed in both cases.

Immunohistochemically, all MASCs showed diffuse and strong staining for CK7, CK8, S100, mammaglobin (secretory material was also positive), STAT5a (signal transducer and activator of transcription 5a) and vimentin (Fig. 2 g, h). Stains for p63 protein and DOG1 were negative in all cases.

### Molecular genetic findings

**Fig. 3:**
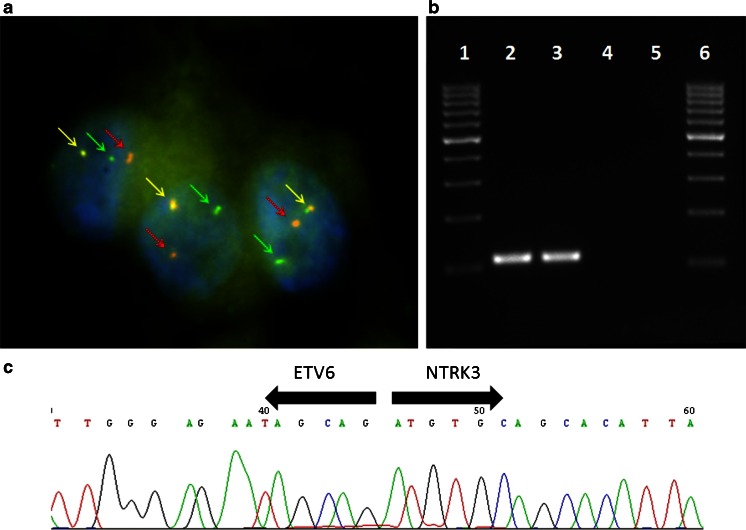
**a** Fluorescent in situ hybridization with ETV6 (12p13) break apart probe. Nuclei with split *red and green signals* indicate ETV6 break. Chromosomes with normal ETV6 gene show *yellow signal* (*overlapping green and red*); **b** expression of the ETV6-NTRK3 fusion transcript by reverse transcription PCR; **c** sequence analysis of the ETV6-NTRK3 fusion transcript. *Arrows* indicate translocation break point

The samples of all seven cases of MASC were analyzed by FISH, and six of seven cases showed ETV6 rearrangement (Fig. 3). In case 1, the cellular material was very limited (cystic tumour with delicate cellular lining) and insufficient for analysis. However, the tumour revealed morphological and immunohistochemical features typical of MASC, and hence, it was finally included in the study. Five AciCC served as negative controls and did not show *ETV6* gene rearrangement (data not shown). The positive control, breast secretory carcinoma, demonstrated *ETV6* gene rearrangement (data not shown).

In all seven cases of MASC, RT-PCR was performed, and in three cases, as well as in the positive control (breast secretory carcinoma, data not shown), *ETV6*-*NTRK3* fusion transcripts were found (Fig. 3).

## Discussion

According to the 2005 WHO Classification of Head and Neck Tumours [2], the group of malignant epithelial salivary gland tumours contains many heterogeneous entities. The histomorphological classification of these tumours is complex, and their clinical behaviour is not completely elucidated, partly because they are so rare. Some entities, such as adenocarcinoma/cystadenocarcinoma NOS, might encompass subtypes still to be discovered by molecular analysis. Careful histomorphological examination of cases that did not entirely fulfil the criteria of one given entity, in conjunction with a typical pattern of expression of immunohistochemical markers, enabled Skálová et al. [26] to define mammary secretory analogue carcinoma (MASC) as a new entity. As a consequence, the recognition and differentiation of MASC from other primary salivary gland tumours are essential in order to clarify its histomorphological features and biological behaviour.

Inspired by the original report and reports from other groups [9, 13, 15, 18, 26], we reviewed primary salivary gland tumours diagnosed in our department and identified seven tumours which met the criteria for MASC. We collected all available clinico-pathological and follow-up data. All cases diagnosed upon revision as MASCs had been diagnosed histologically as malignancy, as adenocarcinoma/cystadenocarcinoma NOS (in three cases), AciCC (two cases) and MEC and SDC (one case each). Fine needle aspiration biopsy (FNAB) results were available in five of seven cases. Two cases had been diagnosed as benign (cyst and adenoma) and in three as malignant lesion.

On cytological smears, MASCs have been reported as variably cellular and with two different architectural patterns: 1) tissue fragments with isomorphic cells arranged in a sheet-like or papillary configuration and 2) dispersed and dissociated cells. Cells contained abundant vacuolated granular and sometimes vacuolated cytoplasm [6, 14, 22, 23]. Nuclear atypia was mild to moderate. Mucin was present, sometimes abundant or absent. MASC cytology represents considerable overlap with other tumours such as AciCC, MEC, SDC and oncocytoma [14] and in the differential diagnosis of low-grade salivary gland neoplasms MASC should be included.

The differential diagnosis of MASC should include AciCC, adenocarcinoma NOS, cystadenocarcinoma and low-grade mucoepidermoid carcinoma. One of our cases (no. 5) was originally diagnosed as salivary duct carcinoma (SDC) due to high-grade transformation prevalent in its morphology [25]. SDC as MASC mimic has not been reported before. Morphologically, the HG component of MASC in both our cases was composed of anaplastic cells with abundant cytoplasm and large polymorphous nuclei arranged in solid structures with focal comedo-like necrosis. In addition, the tumours showed high mitotic activity and invasion of stroma and of peripheral nerves. The high-grade component did not contain colloid- or protein-like material, but the presence of ETV6 rearrangement was confirmed. Immunohistochemistry may be useful to differentiate MASC with high-grade transformation from SDC, which, in contrast to MASC, typically shows expression of androgen receptor or HER-2/neu but not of S100 protein.

The other MASC case was previously diagnosed as mucoepidermoid carcinoma (MEC) with intermediate differentiation, mostly due to focal but unequivocal PAS-positive mucinous differentiation. This feature and variable expression of myoepithelial markers (HMWCK, p63 and CD10) make MEC an important differential diagnosis from MASC [10, 17]. However, the basal/myoepithelial markers (calponin, p63 and CD10) are usually diffusely and strongly expressed in MEC, while weak and focal in MASC [17]. Additionally, lack of squamoid areas with intercellular bridges and/or basal-like intermediate cells supports a diagnosis of MASC. Moreover, MEC often (in 38 to 81 % of cases) harbours a t(11;19) translocation resulting in *CRTC1*-*MAML2* fusion transcript [12, 20]. This differs from MASC, which tends to have the t(12;15)(p13;q25) translocation resulting in the *ETV6*-*NTRK3* fusion transcript.

The most common mimic of MASC is zymogen granule-poor AciCC [13, 18]. We also found two cases (out of 17, 12 %) formerly diagnosed as AciCC: one with papillary cystic (case 3) and the other with macrocystic (case 4) growth pattern. AciCC is characterized by a wide variety of architectural patterns, some of which (microcystic, follicular and papillary cystic) need to be differentiated from MASC. Strong and diffuse S100 protein expression and positive mammaglobin staining should favour a diagnosis of MASC [4, 21].

Adenocarcinoma/cystadenocarcinoma not otherwise specified (ANOS) is a poorly defined entity of otherwise unclassifiable salivary gland carcinoma. Its diagnosis should be made by exclusion of other salivary gland carcinoma types. The differentiation from MASC requires evidence of the *ETV6*-*NTRK3* translocation through which many cases diagnosed as ANOS were reclassified as MASC [3, 5, 9, 13, 15, 24, 29]. Our series contained 14 cases of ANOS, three of which (21 %) were reclassified as MASC.

We performed both FISH and RT-PCR for molecular genetic analysis. In our study, on FISH, all but one case (6/7) was positive for ETV6 gene rearrangement. In the cystic tumour with delicate cellular lining (case 1), the neoplastic material was very limited and, thus, insufficient for analysis. By RT-PCR, only three out of seven cases were positive for the t(12,15) (*ETV6*-*NTRK3*) fusion transcript. Petersson et al. proposed as possible explanation for negative RT-PCR results a different fusion partner for the *ETV6* gene [22]. In haematopoietic malignant disorders, *ETV6* fusions other than with *NTRK3* have been described with *ABL1*, *RUNX1* or *FLT3* [9]. Moreover, the *ETV6*-*NTRK3* fusion is not found in 100 % of secretory carcinomas of the breast [22].

The majority of MASC arose in the parotid gland, followed by the oral cavity (lip, soft palate and buccal mucosa) and submandibular gland [5, 26]. Of our cases, all but one (85 %) developed in the parotid gland. The remaining tumour arose in a small salivary gland of the hard palate. The male predominance in our series (2.5:1) is more prominent than in other reports which found MASC to be only slightly more common in males (53 %) [3]. Age varied widely in our cases from 17 to 73 years (median 51.4) corresponding to earlier data (range from 14 to 78 years) [3]. The size of MASC ranged from 0.2 to 5.5 cm [3, 6, 9, 13–15, 18, 19, 22, 26, 29]. In our series, the smallest tumour (2.0 cm) was located in the hard palate (case 7), whereas others ranged from 2.2 to 4 cm. This is consistent with data from the literature in that MASC in the oral cavity tends to be smaller (mean 0,9 cm) than in the parotid gland (mean 2, 2 cm) [3].

The limited number of cases of MASC with full clinical correlation and follow-up data precludes assessment of its prognosis and response to treatment. Although MASC is currently treated as a low-grade carcinoma with overall favourable prognosis, it has potential for regional lymph node metastasis. In cases with positive surgical margins [9, 26], the tumour often recurs locally, and therefore, adjuvant radiotherapy is recommended. Two of our patients with MASC with high-grade transformation (cases 5 and 6) died of neoplastic disease, one with distant metastasis 20 and 79 months after primary surgery. MASC has a capacity for an aggressive course, and the *ETV6*-*NTRK3* translocation might provide a potential therapeutic target [9].

In conclusion, MASC is a morphologically and molecularly well-defined salivary gland neoplasm. MASC may share microscopic features with AciCC, adenocarcinoma/cystadenocarcinoma NOS and low-grade MEC. In rare cases, MASC with high-grade transformation may morphologically mimic high-grade salivary gland malignancies, such as salivary duct carcinoma.
